# Supplementary material for: Development of multiplex real-time PCR for simultaneous detection of common fungal pathogens in invasive mycoses
Source: PeerJ. 2024 Oct 17;12:e18238. doi: 10.7717/peerj.18238 (PMC11491059; doi:10.7717/peerj.18238)
Supplement: Supplemental Information 1 [file peerj-12-18238-s001.docx]

Table 1: List of organisms and their source used for the analytical specificity evaluation.

| Organism | Source | No. of strains (*n*=65) |
| --- | --- | --- |
| *Aspergillus fumigatus* | ATCC, USA & USM, Malaysia | 3 |
| *Aspergillus terreus* | ATCC, USA & USM, Malaysia | 3 |
| *Candida albicans* | ATCC, USA & USM, Malaysia | 14 |
| *Candida glabrata* (now classified as *Nakaseomyces glabratus)* | USM, Malaysia | 8 |
| *Candida tropicalis* | USM, Malaysia | 1 |
| *Candida parapsilosis* | USM, Malaysia | 1 |
| *Candida krusei* (now classified as *Pichia kudriavzevii*) | ATCC, USA | 1 |
| *Candida lusitaniae* (now classified as *Clavispora lusitaniae*) | USM, Malaysia | 1 |
| *Candida dubliniensis* | USM, Malaysia | 1 |
| *Cryptococcus neoformans* | USM, Malaysia | 1 |
| *Rhodotorula mucilaginosa* | USM, Malaysia | 1 |
| *Trichosporon mycotoxinovorans* | USM, Malaysia | 1 |
| *Aspergillus flavus* | USM, Malaysia | 1 |
| *Aspergillus niger* | USM, Malaysia | 1 |
| *Aspergillus nidulans* | USM, Malaysia | 1 |
| *Aspergillus lentulus* | USM, Malaysia | 2 |
| *Exophiala dermatitidis* | USM, Malaysia | 1 |
| *Fusarium solani* | USM, Malaysia | 1 |
| *Geotrichum candidum* | USM, Malaysia | 1 |
| *Lomentospora prolificans* | USM, Malaysia | 1 |
| *Microsporum canis* | USM, Malaysia | 1 |
| *Microsporum gypseum* | USM, Malaysia | 1 |
| *Meyerozyma guillermondii* | USM, Malaysia | 1 |
| *Mucor* spp. | USM, Malaysia | 1 |
| *Paecilomyces aurantiacus* | USM, Malaysia | 1 |
| *Paecilomyces variotii* | USM, Malaysia | 1 |
| *Rhizopus microsporus* | USM, Malaysia | 1 |
| *Sporothrix schenckii* | USM, Malaysia | 1 |
| *Talaromyces marneffei* | USM, Malaysia | 1 |
| *Trichophyton rubrum* | USM, Malaysia | 1 |
| *Burkholderia pseudomallei* | USM, Malaysia | 1 |
| *Escherichia. coli* | ATCC, USA | 1 |
| *Enterococcus faecalis* | ATCC, USA | 1 |
| *Klebsiella pneumoniae* | ATCC, USA | 1 |
| *Neisseria meningitis* | ATCC, USA | 1 |
| *Staphylococcus aureus* | ATCC, USA | 1 |
| *Staphylococcus epidermidis* | ATCC, USA | 1 |
| *Salmonella enterica* subsp. *enterica* ser. Typhi | ATCC, USA | 1 |
| *Pseudomonas aeruginosa* | ATCC, USA | 1 |
| *Vibrio parahaemolyticus* | ATCC, USA | 1 |
