# Supplementary material for: Development of multiplex real-time PCR for simultaneous detection of common fungal pathogens in invasive mycoses
Source: PeerJ. 2024 Oct 17;12:e18238. doi: 10.7717/peerj.18238 (PMC11491059; doi:10.7717/peerj.18238)
Supplement: Supplemental Information 2 [file peerj-12-18238-s002.docx]

**Supplemental Information**

Table S2. (a)Homo- and heterodimerizations between primers designed in this study.

| Oligonucleotides interaction | | *bgt1* | | *benA* | | *ITS2* | | *LEU2* | | *glmM* | |
| --- | --- | --- | --- | --- | --- | --- | --- | --- | --- | --- | --- |
|  |  | F | R | F | R | F | R | F | R | F | R |
| *bgt1* | F | -3.14 | -6.69 | -11.27 | -4.67 | -5.50 | -6.60 | -5.02 | -5.02 | -5.09 | -7.06 |
|  | R | -6.69 | -10.36 | -6.24 | -4.87 | -5.09 | -3.61 | -4.89 | -3.30 | -10.36 | -6.75 |
| *benA* | F | -11.27 | -6.24 | -6.50 | -5.02 | -3.89 | -5.02 | -8.09 | -8.09 | -5.02 | -6.21 |
|  | R | -4.67 | -4.87 | -5.02 | -4.64 | -3.54 | -5.99 | -8.09 | -8.09 | -6.12 | -8.64 |
| *ITS2* | F | -5.50 | -5.09 | -3.89 | -3.54 | -3.89 | -4.95 | -3.90 | -3.89 | -5.09 | -10.21 |
|  | R | -6.60 | -3.61 | -5.02 | -5.99 | -4.95 | -6.30 | -3.78 | -4.25 | -5.02 | -5.12 |
| *LEU2* | F | -5.02 | -4.89 | -8.09 | -8.09 | -3.90 | -3.78 | -6.84 | -3.89 | -8.09 | -6.69 |
|  | R | -5.02 | -3.30 | -8.09 | -8.09 | -3.89 | -4.25 | -3.89 | -1.94 | -8.09 | -8.56 |
| *glmM* | F | -5.09 | -10.36 | -5.02 | -5.02 | -5.09 | -5.02 | -8.09 | -8.09 | -10.36 | -6.75 |
|  | R | -7.06 | -6.75 | -6.21 | -8.64 | -10.21 | -5.12 | -6.69 | -8.56 | -6.75 | -3.61 |

Table S2. (b)Homo- and heterodimerizations between primers and probes designed in this study.

| Oligonucleotides interaction | | *bgt1* | *benA* | *ITS2* | *LEU2* | *glmM* |
| --- | --- | --- | --- | --- | --- | --- |
|  |  | Probe | Probe | Probe | Probe | Probe |
| *bgt1* | F | -8.16 | -6.69 | -7.81 | -5.02 | -6.21 |
|  | R | -6.75 | -9.85 | -6.75 | -6.75 | -5.09 |
| *benA* | F | -8.09 | -9.31 | -7.71 | -6.50 | -6.14 |
|  | R | -12.76 | -7.07 | -7.71 | -5.02 | -6.14 |
| *ITS2* | F | -7.04 | -7.42 | -5.49 | -3.61 | -4.74 |
|  | R | -6.90 | -4.64 | -3.61 | -3.61 | -2.91 |
| *LEU2* | F | -4.38 | -3.89 | -3.42 | -3.07 | -5.36 |
|  | R | -4.67 | -5.24 | -5.49 | -4.67 | -2.91 |
| *glmM* | F | -8.09 | -5.19 | -9.19 | -6.75 | -6.14 |
|  | R | -9.28 | -6.68 | -10.29 | -6.75 | -10.88 |

Table S2. (c)Homo- and heterodimerizations between probes designed in this study.

| Oligonucleotides interaction | | *bgt1* | *benA* | *ITS2* | *LEU2* | *glmM* |
| --- | --- | --- | --- | --- | --- | --- |
|  |  | Probe | Probe | Probe | Probe | Probe |
| *bgt1* | Probe | -4.67 | -5.12 | -6.75 | -11.42 | -8.51 |
| *benA* | Probe | -5.12 | -6.34 | -6.68 | -7.07 | -5.37 |
| *ITS2* | Probe | -6.75 | -6.68 | -6.75 | -11.42 | -5.37 |
| *LEU2* | Probe | -11.42 | -7.07 | -11.42 | -4.67 | -6.21 |
| *glmM* | Probe | -8.51 | -5.37 | -5.37 | -6.21 | -5.7 |
