# Supplementary material for: Development of multiplex real-time PCR for simultaneous detection of common fungal pathogens in invasive mycoses
Source: PeerJ. 2024 Oct 17;12:e18238. doi: 10.7717/peerj.18238 (PMC11491059; doi:10.7717/peerj.18238)
Supplement: Supplemental Information 5 [file peerj-12-18238-s005.docx]

Table S3 (a) Optimization of *bgt1* primer concentrations.

| **Primer concentration** | **Mean Cq** | **Cq difference (Cq_any_ – Cq_lowest_)** |
| --- | --- | --- |
| 100 nM | 22.17 | 2.09 |
| 200 nM | 22.58 | 2.50 |
| 300 nM | 22.47 | 2.39 |
| 400 nM | 22.05 | 1.97 |
| 500 nM | 20.08 | 0 |
| 600 nM | 22.51 | 2.43 |

Table S3 (b) Optimization of *benA* primer concentrations.

| **Primer concentration** | **Mean Cq** | **Cq difference (Cq_any_ – Cq_lowest_)** |
| --- | --- | --- |
| 100 nM | 22.94 | 1.66 |
| 200 nM | 22.14 | 0.86 |
| 300 nM | 21.69 | 0.41 |
| 400 nM | 21.69 | 0.41 |
| 500 nM | 21.28 | 0 |
| 600 nM | 21.58 | 0.30 |

Table S3 (c) Optimization of ITS2 primer concentrations.

| **Primer concentration** | **Mean Cq** | **Cq difference (Cq_any_ – Cq_lowest_)** |
| --- | --- | --- |
| 100 nM | 23.34 | 0.92 |
| 200 nM | 24.85 | 2.43 |
| 300 nM | 24.98 | 2.56 |
| 400 nM | 24.84 | 2.42 |
| 500 nM | 22.42 | 0 |
| 600 nM | 25.52 | 3.10 |

Table S3 (d) Optimization of *LEU2* primer concentrations.

| **Primer concentration** | **Mean Cq** | **Cq difference (Cq_any_ – Cq_lowest_)** |
| --- | --- | --- |
| 100 nM | 30.28 | 2.32 |
| 200 nM | 30.04 | 2.08 |
| 300 nM | 28.97 | 1.01 |
| 400 nM | 28.37 | 0.41 |
| 500 nM | 27.96 | 0 |
| 600 nM | 28.41 | 0.45 |

Table S4 (a) Optimization of *bgt1* probe concentrations.

| **Probe concentration** | **Mean Cq** | **Cq difference (Cq_any_ – Cq_lowest_)** |
| --- | --- | --- |
| 50 nM | 24.17 | 0.29 |
| 100 nM | 26.44 | 2.56 |
| 150 nM | 24.12 | 0.24 |
| 200 nM | 23.88 | 0 |
| 250 nM | 24.04 | 0.16 |

Table S4 (b) Optimization of *benA* probe concentrations.

| **Probe concentration** | **Mean Cq** | **Cq difference (Cq_any_ – Cq_lowest_)** |
| --- | --- | --- |
| 50 nM | 21.23 | 0.59 |
| 100 nM | 21.06 | 0.42 |
| 150 nM | 21.48 | 0.84 |
| 200 nM | 21.07 | 0.43 |
| 250 nM | 20.64 | 0 |

Table S4 (c) Optimization of ITS2 probe concentrations.

| **Probe concentration** | **Mean Cq** | **Cq difference (Cq_any_ – Cq_lowest_)** |
| --- | --- | --- |
| 50 nM | 21.85 | 0.98 |
| 100 nM | 21.33 | 0.46 |
| 150 nM | 21.08 | 0.21 |
| 200 nM | 21.01 | 0.14 |
| 250 nM | 20.87 | 0 |

Table S4 (d) Optimization of *LEU2* probe concentrations.

| **Probe concentration** | **Mean Cq** | **Cq difference (Cq_any_ – Cq_lowest_)** |
| --- | --- | --- |
| 50 nM | 23.93 | 1.93 |
| 100 nM | 22.62 | 0.62 |
| 150 nM | 22.55 | 0.55 |
| 200 nM | 22.30 | 0.30 |
| 250 nM | 22.00 | 0 |
